# Comparison of Clinical and Dermoscopic Features Between Micronodular and Nodular Basal Cell Carcinoma

**DOI:** 10.1111/ijd.70163

**Published:** 2025-11-26

**Authors:** Giulia Briatico, Camila Scharf, Maria Maddalena Nicoletti, Antonio Petti, Gabriella Brancaccio, Eugenia Veronica Di Brizzi, Elvira Moscarella, Andrea Ronchi, Giuseppe Argenziano

**Affiliations:** ^1^ Dermatology Unit University of Campania Naples Italy; ^2^ Division of Pathology, Department of Mental Health and Preventive Medicine, Luigi Vanvitelli University of Campania Naples Italy

**Keywords:** basal cell carcinoma, dermoscopy, diagnosis

Micronodular basal cell carcinoma (mBCC) is considered histologically more aggressive than nodular BCC (nBCC), with higher risk of subclinical extension and incomplete excision [1]. However, dermoscopic diagnostic criteria for mBCC remain poorly defined. Only few studies, with limited sample sizes, have explored this subtype [1, 2]. We aimed to compare clinical, dermoscopic, and surgical features of mBCC and nBCC in a large retrospective series.

We retrospectively reviewed 105 mBCC and 102 nBCC with available high‐quality clinical and dermoscopic images. Images were acquired with Canon and dermlite foto II. Only histologically confirmed cases were included. Mixed BCCs were excluded. Four dermatologists (> 5 years dermoscopy experience) blindly scored predefined dermoscopic features according to published criteria [3, 4]. Clinical variables, dermoscopic criteria, margin status, reinterventions, and recurrences were recorded. Statistical comparisons used *χ* ^2^ or Fisher's exact test; *p* < 0.05 was considered significant.

A total of 105 mBCC and 102 nBCC were included. Median age was similar (70 vs. 73 years), with a male predominance of 58% in both groups. The majority of tumors were located on the head and neck (77% of mBCC and 70% of nBCC; *p* = 0.283), most commonly on the nose (26% vs. 29%) and forehead (23% vs. 18%), without significant differences in subsite distribution.

Dermoscopically, mBCC showed a significantly larger vascularized surface, with vessels covering more than half of the lesion in 59.0% compared with 13.7% of nBCC (*p* < 0.001). Branched vessels predominated in mBCC (74% vs. 45%, *p* < 0.001), whereas linear vessels were more common in nBCC (21% vs. 10%, *p* = 0.026). Erosions were less frequent in mBCC (12.4% vs. 24.5%, *p* = 0.024). Other dermoscopic features, including ulceration, pigmentation patterns, white streaks, May‐globules, scales, and milky‐red areas, were not significantly different between groups. A full comparison is provided in (Table 1; Figure 1).

**TABLE 1:** Characteristics of mBCC (micronodular basal cell carcinoma) and nBCC (nodular basal cell carcinoma).

Features	mBCC n/N (%)	nBCC n/N (%)	*p*‐value	Effect size
Ulceration	36/105 (34.3%)	44/102 (43.1%)	0.191	
Erosion	13/105 (12.4%)	25/102 (24.5%)	**0.0242**	OR 0.44 (95% CI 0.21–0.91)
White streaks	18/105 (17.1%)	21/102 (20.6%)	0.526	
May‐globules	10/105 (9.5%)	16/102 (15.7%)	0.181	
Scales	32/105 (30.5%)	34/102 (33.3%)	0.659	
Milky‐red areas	21/63 (33.3%)	29/102 (28.4%)	0.506	
Pigmented structures (any)	55/105 (52.4%)	50/102 (49.0%)	0.629	
Pigmentation > 50% area	20/105 (19.0%)	18/102 (17.6%)	0.795	
Vessels > 50% area	62/105 (59.0%)	14/102 (13.7%)	**< 0.001**	OR 9.06 (95% CI 4.57–17.98)
Vessels subtype				
Branched	78	46	**< 0.001**	OR 3.52 (95% CI 1.96–6.32).
Linear	10	21	**0.026**	OR 0.41 (95% CI 0.18–0.91)
Dotted	0	1	0.493	
Mixed	6	8	0.542	
Absent	11	26	**0.012**	OR 0.38 (95% CI 0.18–0.82)

*Note:* Bold indicates statistically significant values.

**FIGURE 1:**
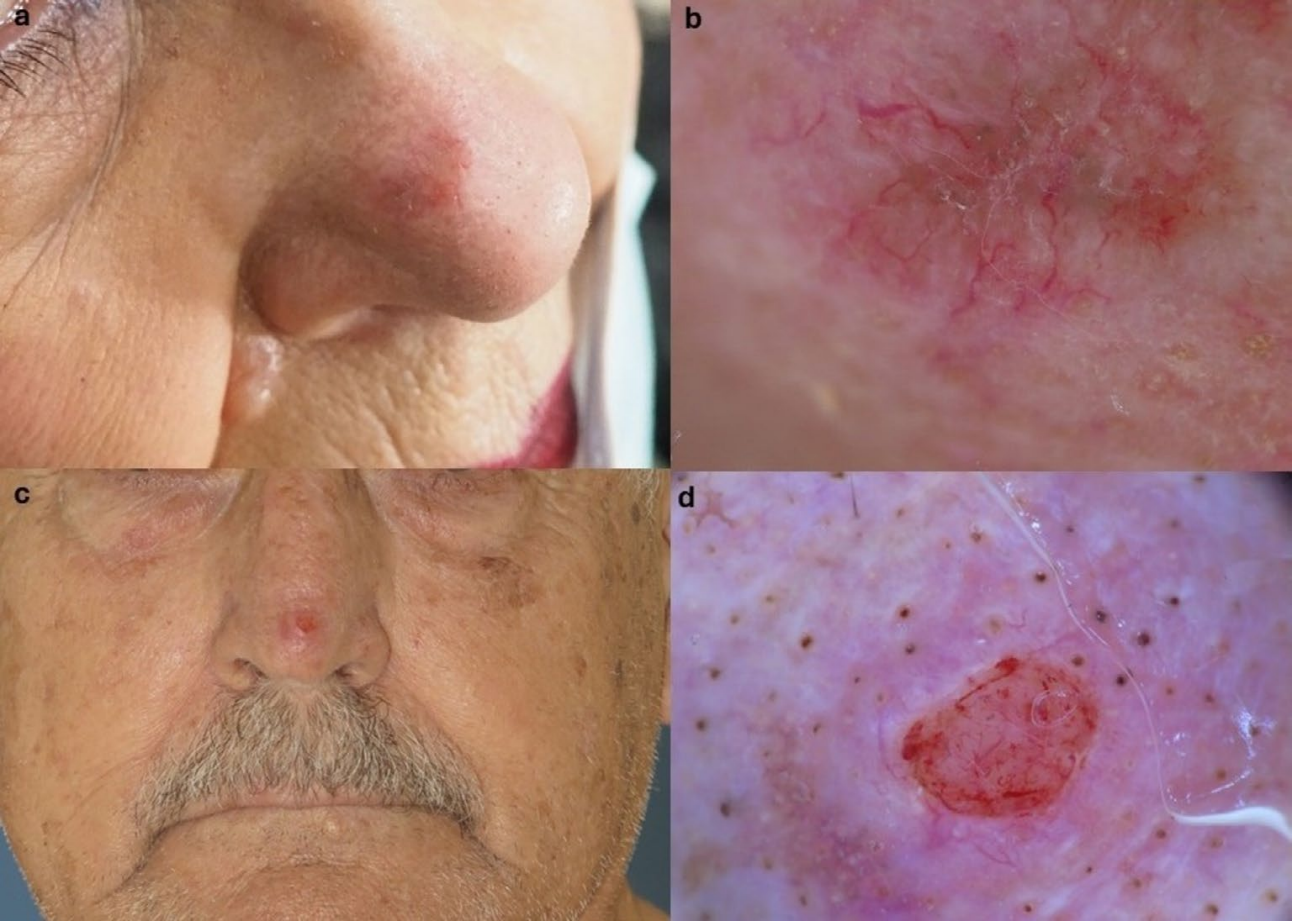
Comparison of two basal cell carcinomas on the nose. On a–b, micronodular BCC appears as a not well‐defined plaque with branched vessels. On c–d, nodular BCC appears as a circumscribed nodule with ulceration. Vessels in this case are not prominent.

Histopathological assessment showed that positive margins were more frequent in mBCC (8% vs. 2%, *p* = 0.053). Local relapses occurred only in the mBCC group (two cases vs. none in nBCC, *p* = 0.241) after mean follow up of 2 years.

Our findings demonstrated that mBCC, compared with nBCC, showed markedly greater vascularization with branched vessels and fewer erosions, while pigmentation patterns did not differ significantly. These dermoscopic clues may support clinical suspicion of mBCC, a subtype usually associated with positive margins and potential relapse. Although differences in outcomes did not reach statistical significance, the trend toward incomplete excision underscores the need for careful surgical management. Previous work has highlighted the aggressive nature of mBCC and the difficulty in its recognition [1, 2], but dermoscopy has rarely been systematically assessed. Our study, to our knowledge, represents the largest dermoscopic analysis of mBCC reported so far. The results align with prior descriptions of vascular patterns in aggressive BCC subtypes [3, 4, 5].

Limitations include retrospective design and exclusion of other histologic subtypes (e.g., infiltrative, morpheaform). Another limit is the brief follow up (2 years) for evaluating relapses. In conclusion, micronodular BCC presented dermoscopically with extensive branched vascularization and fewer erosions compared with nodular BCC. Awareness of these features may assist in recognition and guide surgical planning.

## Consent

Patients gave informed consent to use their images.

## Conflicts of Interest

The authors declare no conflicts of interest.

## Data Availability

The data that support the findings of this study are available on request from the corresponding author. The data are not publicly available due to privacy or ethical restrictions.
